# Pain—novel targets and new technologies

**DOI:** 10.3389/fphar.2014.00211

**Published:** 2014-09-16

**Authors:** Susan Hua, Peter J. Cabot

**Affiliations:** ^1^The School of Biomedical Sciences and Pharmacy, The University of NewcastleCallaghan, NSW, Australia; ^2^School of Pharmacy, The University of QueenslandBrisbane, QLD, Australia

**Keywords:** pain, analgesics, novel strategies, therapeutic target, targeted drug delivery

Pain is a major health problem that significantly affects the quality of life of patients. It has a significant impact on both the sufferers and the broader community, imparting high health costs, and economic loss to society. The consensus among clinicians and researchers worldwide is that current strategies for the treatment of pain are inadequate. These inadequacies are even greater when chronic pain, which often accompanies chronic illnesses such as arthritis or nerve injury, is involved. Despite major advances in treatment strategies over the last two decades, pain management still remains a major challenge in arthritis; and even with treatment with current therapies, many patients still experience moderate-to-severe pain (Stein and Baerwald, 2013). Patients with rheumatoid arthritis report pain management as their highest priority (Whittle et al., 2013), and osteoarthritis is the leading cause of pain and physical disability in the elderly (Stein and Baerwald, 2013). The burden of disease, especially with osteoarthritis, is growing in relation to the aging population and the increasing levels of obesity in the world population. Similar concerns are growing for other types of pain.

Current analgesics for persistent pain are relatively ineffective, are associated with significant adverse effects or abuse liability, and do not reduce pain in all treated individuals (Woolf, 2010). Opioids (e.g., morphine, codeine, oxycodone) are currently one of the most potent groups of analgesics used clinically (Iwaszkiewicz et al., 2013), with prescriptions increasing by 50% over the past 10 years for chronic, non-cancer pain (Waterman, 2013). However there is clear evidence that as opioid prescription rate rises, there is a corresponding increase in opioid overdose deaths, misuse and addiction, with these adverse effects attributed to their agonist effects on central opioid receptors—causing dependence, tolerance, sedation, and respiratory depression (Hua and Cabot, 2010; Waterman, 2013). Non-steroidal and steroidal anti-inflammatory drugs have serious side effects such as gastric erosions, ulcer formation, bleeding, hypersensitivity reactions, cardiovascular toxicity, renal toxicity, and hepatotoxicity (Warner and Mitchell, 2008; Stein et al., 2009). In addition, they are also not peripherally selective thereby causing a range of central adverse effects (Stein et al., 2009). Over the past 20 years, most analgesic development activity have been limited to reformulation of opioids, production of new cyclooxygenase (COX) inhibitors, amine reuptake inhibitors and anticonvulsants, and introduction of topical local anesthetics—all of these act on well-established targets (Woolf, 2010). Therefore, there is an obvious clinical need to introduce more effective and safe analgesics, suitable for chronic administration.

The problem of clinical pain management is complex and far-reaching, as it encompasses many different types of pain, such as arthritic, musculoskeletal, neuropathic, and visceral pain. Our increasing understanding of the neurobiology of pain further supports that a “one size fits all” policy is not appropriate for the way we treat pain across different pathological pain conditions as well as for individuals with the same underlying condition. Pain is commonly a manifestation of a range of multiple, sometimes irreversible, abnormalities in the functioning of the nervous system. In many cases the problem is the persistent amplification of sensory signals and generation of spontaneous activity in the nervous system, which occurs in conditions such as fibromyalgia, neuropathic pain, irritable bowel syndrome, and headaches (Woolf and Salter, 2000; Latremoliere and Woolf, 2009). The complexity and heterogeneity of pain should be appreciated. Complex interplay of processes operating at multiple peripheral and central sites are involved in initiating or sustaining pain, with each mechanism involving many unique or similar targets (Woolf, 2010).

The driving force for the successful translational development of novel analgesics requires the collaboration of experts in the field of basic pain science, pharmaceutics and clinicians specializing in pain management. Drug delivery and targeting is now recognized as the key to effective development of many novel and existing therapeutics to enable optimal therapeutic use of such molecules, as many drugs are severely compromised by significant obstacles to delivery *in vivo* and by toxic adverse effects (Hua and Wu, 2013). Drug delivery systems have been used in pain therapies to improve toxicity or side effect profiles by targeted delivery to specific sites in the body, increase drug bioavailability, and providing prolonged drug release (Hua and Cabot, 2013; Hua and Wu, 2013). There is also a need for detailed phenotyping of animal models of pain and evaluation of whether the models are appropriate surrogates for human pain syndromes. In cases where there is no good rodent model of the disease, it may be better to model pain mechanisms, such as peripheral sensitization or ectopic excitability in nociceptors using electrophysiology (Woolf, 2010). It may be very likely that a single pain-relieving magic bullet simply does not exist, and instead our focus may need to turn to multiple targeted treatments and/or synergistic therapies that are aimed at the specific mechanisms responsible.

This Research Topic focuses on articles that discuss the mechanisms of various types of pain as well as identifying potential novel targets and new technologies for the development of innovative therapeutic strategies for the treatment of pain.

## Conflict of interest statement

The authors declare that the research was conducted in the absence of any commercial or financial relationships that could be construed as a potential conflict of interest.
